# Rainfed winter wheat cultivation in the North German Plain will be water limited under climate change until 2070

**DOI:** 10.1186/s12302-015-0061-6

**Published:** 2015-11-10

**Authors:** Nikolai Svoboda, Maximilian Strer, Johannes Hufnagel

**Affiliations:** Institute of Land Use Systems, Leibniz Centre for Agricultural Landscape Research, Eberswalder Straße 84, 15374 Müncheberg, Germany

**Keywords:** Summer rainfall, Growing period, Resource efficient production systems

## Abstract

**Background:**

We analysed regionalised ECHAM6 climate data for the North German Plains (NGP) in two time slots from 1981 to 2010 and 2041 to 2070.

**Results:**

The annual mean temperature will increase significantly (by about 2 °C) that will result in shorter growing periods since the sum of degree days until harvest will be reached earlier. Even if the amount of total precipitation does not change there appears to be a shift towards increased winter precipitation and thus noticeable reduced summer precipitation.

**Conclusions:**

Through the example of winter wheat we show a future limitation of water availability if yields are to be maintained or even increase.

## Background

Water is fundamental to plant growth, so the impact of climatic water availability on crop production is significant. Extreme yield drops in Europe in 2003 (loss of 13 billion Euros) were associated with an environmental temperature increase of nearly 6 °C above the long-term mean and below average precipitation of approximately 300 mm [17]. Many authors [9, 11, 12] show there is a general increase in winter precipitation, visible in predicted climate data. Meinke et al. [12] show an increase in winter precipitation with regional climate models, for North Germany, of +22 %, but a decrease in summer of −17 %. Thus, we could expect reduced summer rainfall and consecutively increased risk of yield losses due to increased water deficit of field crops. Aim of this study is to evaluate if there may arise serious problems and answer the following questions:

1. Is there a relevant change by comparing the status quo with current climate projections?
2. Is there a shift towards winter rainfall in the NGP, and in particular in the study regions, as predicted in the literature?
3. Is there a trend to decreased and less steady rainfall during the summer growing period of winter wheat visible when evaluating current climate projections?

## Methods

### Study area

**Fig. 1:**
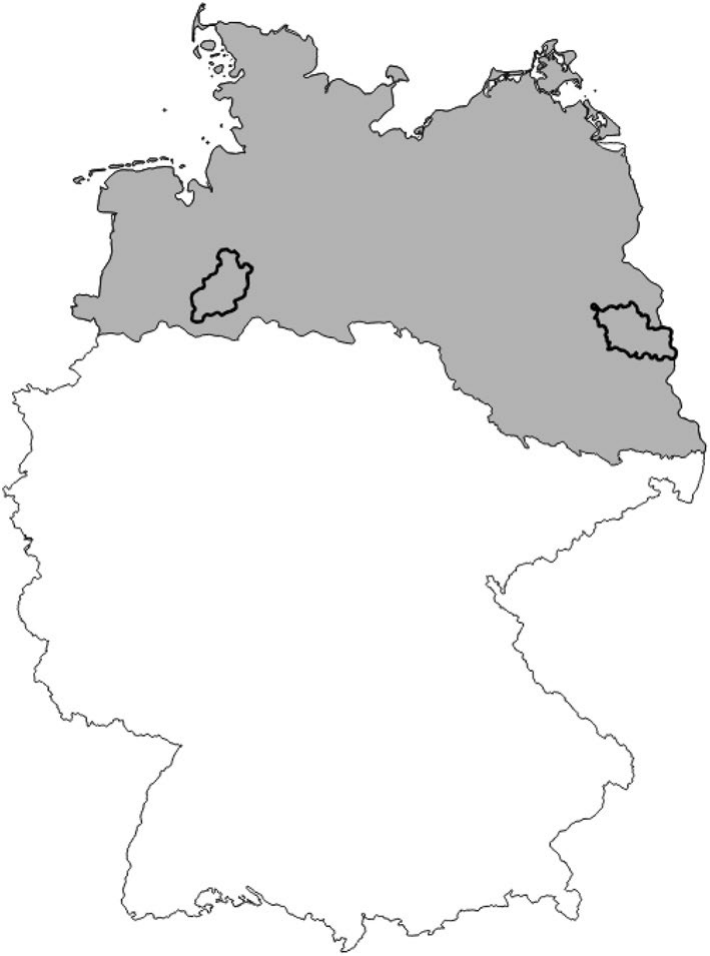
The North German Plain (*grey*) and the study regions Diepholz (*left*) and Oder-Spree (*right*)

**Fig. 2:**
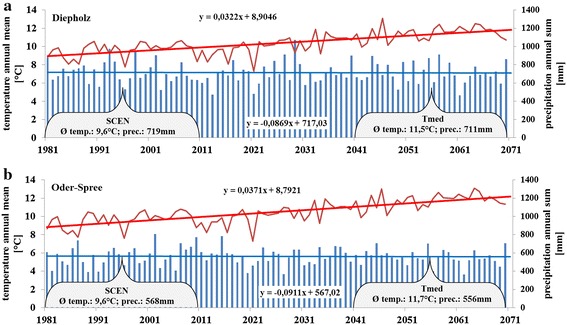
Mean annual temperature (temp.) and sum of annual precipitation (prec.) of both study areas Diepholz (**a** *top*) and Oder-Spree (**b** *bottom*). Mean temperature and precipitation during the investigation period SCEN (1981–2010) as well as *T*_med_ (2041–2070) is given

The North German Plain (NGP) covers the administrative units of Schleswig–Holstein, Mecklenburg Vorpommern, Lower Saxony, Brandenburg and parts of Saxony-Anhalt. As described in Dickinson [4] most of the area is less than 100 m in altitude, and only its zones of low hills reach more than 200 m. Surface deposits are the results of glaciation. The general climate follows a gradient of increasing continentality from west (oceanic) to east (sub-continental). The mean annual temperature is comparable across the NGP but the western part has a temperature range, from annual minimum to annual maximum, of 16.4 °C and the eastern part a range of 18.5 °C. The western part of the NGP has a precipitation of 600–800 mm per year, while the eastern part has a smaller total of 500–600 mm [4]. Main field crops in terms of acreage in the NGP are winter wheat, winter rape, silage maize and winter rye. In the present study, Diepholz (DH) as the most western and Oder-Spree (OS) as the most eastern regions were investigated (Fig. 1). DH has a long-term (1981–2010) mean temperature of 9.6 °C and 719 mm of precipitation (Fig. 2a). OS has 9.6 °C and 568 mm in long term (Fig. 2b). In 2003, precipitation in DH was measured at 523 mm and 434 mm in OS, respectively. In DH, 2003 was the year with the lowest precipitation during the observation period (1981–2010).

### Crop

Winter wheat is the most important crop in the NGP and, matching with Boogaard et al. [2], the dominant crop of Europe in terms of acreage. In DH, 16 % of all cropping area is winter wheat (WW). In OS, the share is 7 %. The sowing date (*JD*_s_) is September 15 as common in the NGP. Due to temperature as the main driver for physiological processes [1], the harvest date of winter wheat is essentially determined by cumulated temperature (heat sum), expressed in degree days (DD) [8]. Growth of winter wheat depends strictly on the air temperature [18].

### Modelling the harvest date and growing period

The duration of the growing period (*V*_per_) is determined by:

1 $$V_{\text{per}} = JD_{\text{h}} + \left( {365\frac{1}{4} - JD_{\text{s}} } \right)$$

with *JD*_h_ [day of year (DOY)] and *JD*_s_ [DOY] being the harvest and the sowing date, and $365\frac{1}{4}$ denoting 365 days per year and 366 in the leap year, respectively. The harvest date *JD*_h_ is defined by

2 $$JD_{\text{h}} = i_{{{\text{GDD}} = T_{\text{h}} }} ,$$

with $i_{{{\text{GDD}} = T_{\text{h}} }}$ being the iterator *i* of growing degree days (GDD) reaching the threshold (*T*_h_). Growing degree days is determined as:

3 $${\text{GDD}} = \mathop \sum \limits_{i = 1}^{{{\text{GDD}} = T_{\text{h}} }} \left\{ {\begin{array}{*{20}l} {\left( {T_{{{\text{mean}}_{i} }} - T_{\text{base}} } \right),\quad T_{{{\text{mean}}_{i} }} \ge T_{\text{base}} } \\ {\left( {T_{{{\text{mean}}_{i} }} - T_{\text{base}} } \right) = 0, \quad {\text{else}}} \\ \end{array} } \right. ,$$

where *T*_mean_, *T*_base_, and *T*_h_ are the daily mean daily temperature, base temperature (*T*_base_ = 2.5 °C, [15]: root growth (3 °C) and shoot growth (2 °C)), and threshold temperature as a fit parameter. The same value (*T*_h_ = 2100 °C) was used for both study sites. We determined—based on harvest and sowing date—the vegetation days (*V*_day_) as the number of days with temperatures above base temperature during growing period (*V*_per_). Therefore, we derived the equation

4 $${\text{DD}}_{M} = \mathop \sum \limits_{{j = JD_{\text{s}} }}^{{JD_{\text{h}} }} \left\{ {\begin{array}{*{20}l} {V_{\text{Day}} + 0, \quad T_{{{\text{MAV}}_{j} }} > T_{\text{base}} } \\ {V_{\text{Day}} + 0,\quad {\text{else }}} \\ \end{array} } \right.$$

where *T*_MAV_ denotes the simple moving average of the mean daily temperature given by

5 $$T_{{{\text{MAV}}_{n} }} = \frac{{T_{{{\text{MAV}}_{(n - 2)} }} + T_{{{\text{MAV}}_{(n - 1)} }} + T_{{{\text{MAV}}_{n} }} + T_{{{\text{MAV}}_{(n + 1)} }} + T_{{{\text{MAV}}_{(n + 2)} }} }}{n}$$

Iterators are *j* and *n*.

### Time slots

Time period analysed within this study is from 1981 until 2070. Within this period we selected two representative time slots of 30 years each. First slot is from 1981 to 2010 representing the status quo and delineates the reference period. The second slot is from 2041 to 2070 representing the future. Differences between the time slots indicate a possible climate change.

### Climate—recent climate

Scenario weather data for representative weather stations are available with daily values for the model regions in the NGP. These data are the result of fitting “Statistical regionalization model: STAR” [13] to recent measured data of the appropriate weather stations. STAR scenario data (SCEN: 1981–2010) then match the observed values for each study area in terms like mean monthly precipitation, temperature and solar radiation. To exclude model bias when comparing status quo with future climate data, all following evaluations of the status quo were based on the scenario (SCEN) climate data.

### Climate—climate change (CC) scenarios for future climate prediction

The results for the current condition were compared to projected weather data driven by the output of general circulation models (GCM) run under representative concentration pathway 8.5 (RCP 8.5). Collective climate models were used for analysis and prediction of climate change. Collective climate models include 21 GCM; all were driven by the scenario RCP 8.5. For the present study, we have selected 3 out of 21 GCM on the basis of their temperature gradient: (a) Minimum mean temperature increase (*T*_min_ → INM-CM4, Russia, +1 °C until 2070). (b) Medium mean temperature increase (*T*_med_ → ECHAM6, MPI Hamburg, Germany, +2 °C). (c) Maximum mean temperature increase (*T*_max_ → ACCESS1.0, CSIRO-BOM, Australia, +3 °C). The regionalisation of the GCM output was realised by the STAR model.

First of all, we need to define which aspects of climate change are relevant concerning crop production in general. Thus, in this study, the relevant climate change intends relevant for cropping winter wheat and includes in particular evaluations during the growing period and this period in parts.

Winter rainfall in our context is defined by DIN 4049 where the hydrological year (*H*_a_) runs from 1 November of year one to 31 October of the following year. The winter season includes the months of November to April; the summer season includes the months of May to October. The second benefit is the start and end of hydrological winter (*H*_W_) that reflects start and end of leaching period in the NGP. Calculating this way enables us to analyse the winter rainfall during the typical leaching period and the summer rainfall from the end of the leaching period during summer until the harvest date, respectively.

Since rainfall during the growing period (*P*_veg_) is not a meaningful parameter for analysing possible water deficit of winter wheat, we introduced the precipitation during main growing period (*P*_m-veg_) as a parameter of interest (beginning of possible water deficit due to emptying the soil water storage with the beginning of hydrological summer); *P*_m-veg_ is defined by the amount of precipitation measured from May 1 (assumed end of leaching period due to the beginning of significant transpiration) until harvest date.

### Statistical analysis

All data were evaluated using the R software package R Core Team [16].

## Results

### Model fit

**Fig. 3:**
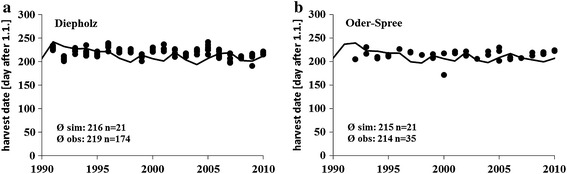
Observed and modelled harvest dates for the study region Diepholz (**a** *left*) and Oder-Spree (**b** *right*) for the time period 1991–2010 (phenological data were provided by the German Weather Service DWD)

Pre-tests showed that, the regional data (scenario) agree with respect to their general temperature trend, their variability and their precipitation with the climate data of the weather stations (observed) in the regions (data not shown). Harvest dates were reasonably well predicted by our simple model. The mean observed harvest dates of the study region Diepholz over 21 years were day 216 while the model underestimates by 3 days. The same good model fit could be shown for the Oder-Spree region where the observed mean harvest date was 214 and the modelled was 214. Annually simulated as well as observed harvest dates are presented in Fig. 3a, b.

### Shift towards winter rainfall

The mean precipitation during the hydrological year (*H*_a_: 1 May until 31 April) for the reference period (SCEN: 1981–2010) is 705 mm for DH and is 566 mm for OS. In the future (2041–2070) precipitation during *H*_a_ ranges between 683 and 711 mm for DH and between 512 and 570 mm for OS depending on the scenario (*T*_min_, *T*_med_, *T*_max_). Thus, there is no significant change in annual precipitation while comparing the reference with the future period. Compared to the very little alteration of total amount of precipitation, standard deviation (as an indicator of constancy) of mean precipitation decreases in DH from 122 (SCEN) to 97 (*T*_med_) and in OS from 101 (SCEN) to 67 (*T*_med_) when comparing recent with future time period (Table 1). Mean precipitation during hydrological winter (*H*_W_) during the SCEN period is 331 mm in DH and 246 mm in OS. Within the future time slot *T*_med_ DH has a mean *H*_W_ precipitation of 387 mm and OS 296 mm, respectively. The share of precipitation during *H*_W_ (*H*_a_/*H*_W_) in the SCEN period is for DH 0.47 and for OS 0.44 and for the future time slot in *T*_med_ for DH 0.54 and for OS 0.53. The scenario *T*_max_ delivered comparable results, while in the *T*_min_ scenario the share ranges between 0.50 (DH) and 0.48 (OS).

**Table 1:** Precipitation in the study regions Diepholz (DH) and Oder-Spree (OS) differentiated according to annual precipitation (1.1.–31.12.), precipitation during hydrological year (1.10.–31.9.), hydrological winter (1.10.–31.4.) and the share of precipitation during hydrological winter (*H*_W_)

	Annual precipitation		Hydrological year		Hydrological winter		Share of *H*_W_	
	SCEN	*T*_med_	SCEN	*T*_med_	SCEN	*T*_med_	SCEN	*T*_med_
DH								
*P* (mm)	709	711	705	711	331	387	0.47	0.54
*SD* (mm)	132	112	122	97	63	50		
OS								
*P* (mm)	572	556	566	556	246	296	0.44	0.53
*SD* (mm)	104	80	101	67	55	43		

*SCEN* represents the recent time period (1981–2010), *T*_med_ the future time period (2041–2070), *SD* is the standard deviation

### Harvest date

**Fig. 4:**
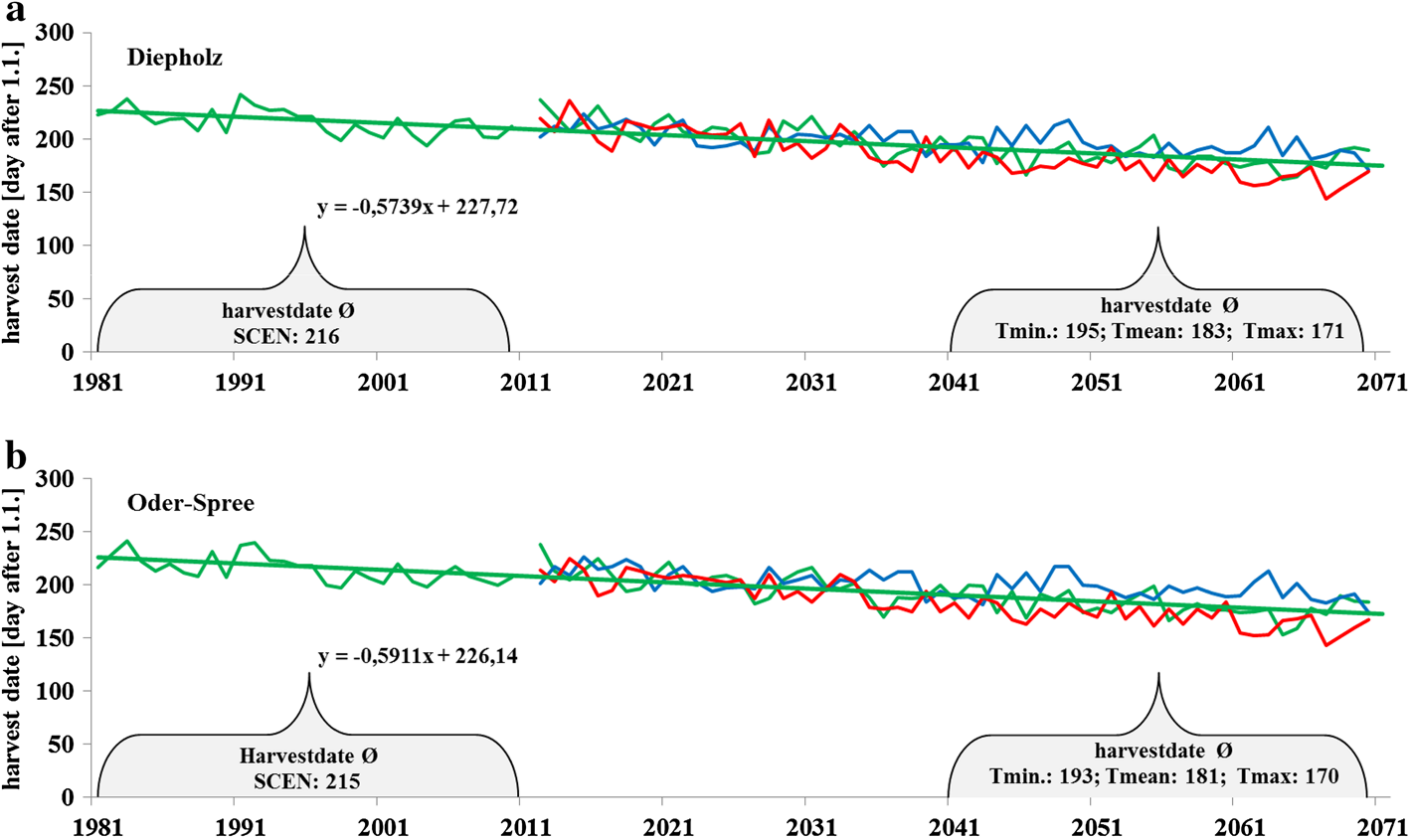
The harvest date as expressed in Julian days after 1 January for the study region Diepholz (**a** *top*) and Oder-Spree (**b** *bottom*). Future harvest dates were calculated on the basis of *T*_min_ (*blue*), *T*_med_ (*green*) and *T*_max_ (*red*) scenario. Linear trend is given for the SCEN and *T*_med_ scenario

The mean harvest date within the SCEN period lies between the 3 and 5 August while for the *T*_med_ period earlier dates between 3 July and 30 June were calculated (Fig. 4a, b). Evaluating *T*_min_, the harvest date is earlier than in SCEN but later than *T*_med_ (13 and 14 July). Much earlier is the *H*_day_ when dealing with the *T*_max_: 19 and 21 June.

### Growing period

Length of the growing period is strongly correlated with the harvest date. The growing period of winter wheat (*V*_per_) during the reference period (SCEN) is 324 days in DH and 323 days in OS while the vegetation days during the *V*_per_ is 267 in DH and 246 in OS (Table 2). Therefore, in DH 57 cold days (days with less than 2.5 °C within the *V*_per_ as an indicator for the frequency of the interruption of biomass accumulation) and in OS 77 cold days were detected during the 1981–2010 period. In the future (2041–2070) period (*T*_med_) the *V*_per_ is shorter by 33 days and 34 days in DH and OS, respectively, when compared to SCEN. The cold days in the *T*_min_ were reduced to 30 (DH) and 42 (OS). Within the *T*_max_ a minimum of cold days of 19 (DH) and 30 (OS) was counted.

**Table 2:** Growing period (days) of winter wheat (*V*_per_) as defined by the delimiters sowing and harvest date for the study regions Diepholz (DH) and Oder-Spree (OS)

	SCEN	*T*_med_
DH		
*V*_per_	324	291
*V*_day_	267	261
OS		
*V*_per_	323	289
*V*_day_	246	246

*SCEN* represents the recent time period (1981–2010), *T*_med_ the future time period (2041–2070)

### Rainfall during main growing period and potential drought

**Fig. 5:**
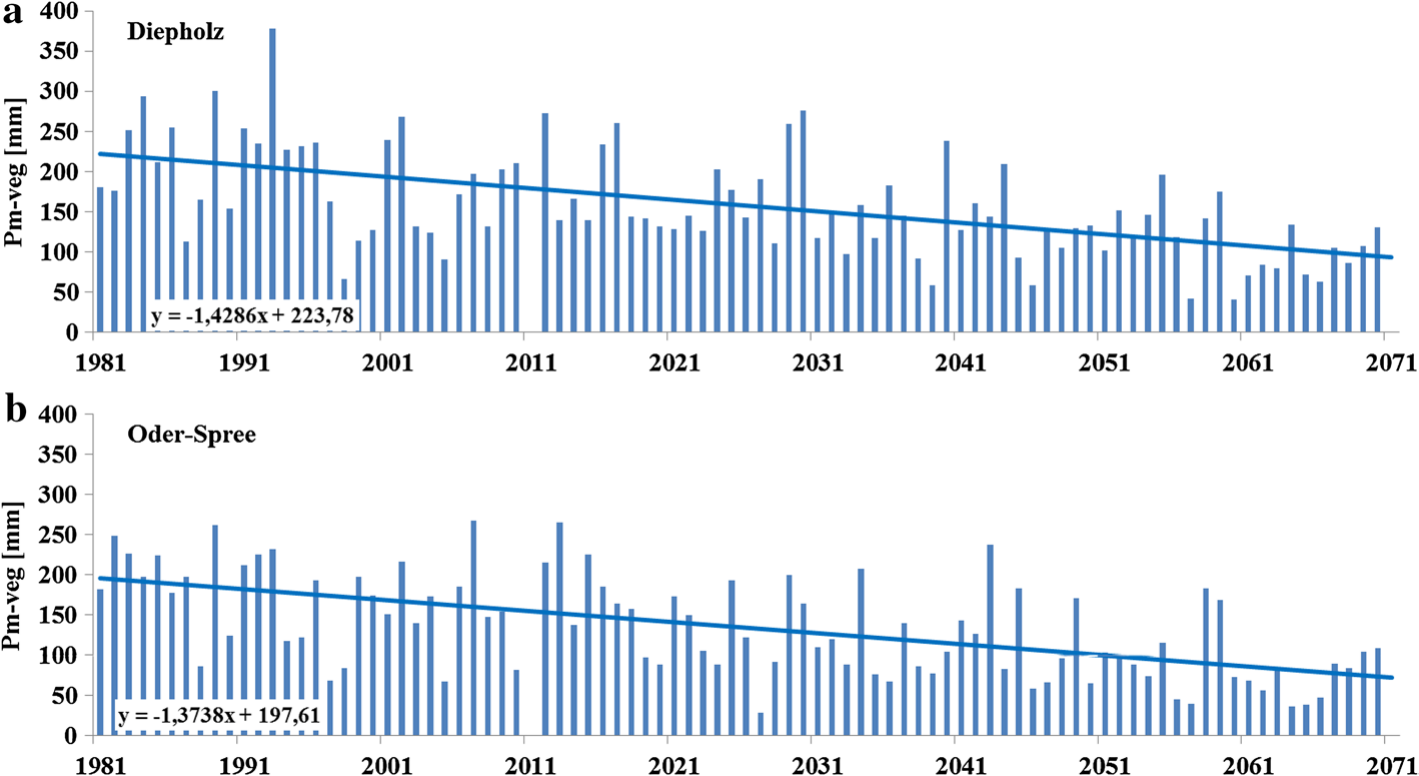
Annual precipitation during main growing period from the beginning of summer until harvest for Diepholz (**a** *top*) and Oder-Spree (**b** *bottom*)

During the main growing period (*P*_m-veg_: 1 May until harvest) 197 mm were measured in DH and 171 mm in OS, Table 3). For the *T*_med_ scenario less rainfall during *P*_m-veg_ 115 to 98 mm was calculated (Fig. 5a, b). Similar results for *P*_m-veg_ can be shown for *T*_min_ (149 mm in DH and 136 mm in OS) and for *T*_max_ (78 mm in DH and 68 mm in OS).

**Table 3:** Precipitation during main growing period (*P*_m-veg_)

	SCEN	*T*_med_
DH		
*P*_m-veg_ (mm)	197	115
OS		
*P*_m-veg_ (mm)	171	98

*SCEN* represents the recent time period (1981–2010), *T*_med_ the future time period (2041–2070)

## Discussion

### Model fit

When comparing the data from the weather stations during the reference period with the modelled STAR outcome no significant differences are noticed. This is in good agreement of Gerstengarbe et al. [7] who compared STAR with the current climatology of selected regions all over Germany. Gallardo et al. [5] show similar results while analysing an ensemble of 15 regional climate models nested into six GCM. They found differences depending on the region and the investigated model. Our simple model for calculating the harvest date reasonably well predicts the mean harvest date over a long period of 30 years. For some years the prediction is less precise. For this reason, we have based all results to the long term.

### Shift towards winter rainfall

The shift towards winter rainfall with +7 % in DH and +9 % in OS is less pronounced than reported in many studies [9, 11, 12]. That may be because of the different period (hydrological vs. calendric) selected on the one hand and the different period of time (1981–2010) in total. Badeck et al. [1] suggested that a fraction of uncertainty may arise due to the time frame analysed. Comparing the mean annual precipitation of calendric against hydrologic year in the present time period, DH shows with 709 mm compared to 711 mm only little difference. However, OS reflects similar results on a lower level (572 to 566 mm). Kozuchowski and Degirmendizc [10] analysed long time weather data in different regions in Poland and found that regional differences are widespread. Following this, it may be possible, that the regions investigated in the present study may have a different shift than the mean of the NGP. Further studies should clarify the situation.

### Harvest date

Patil et al. [14] found evidence that increased temperature led to earlier harvest date; the same effect we discovered for both regions. Depending on the scenario (*T*_min_, *T*_med_, *T*_max_) the harvest date will be three (*T*_min_), five (*T*_med_) or six (*T*_max_) weeks earlier than today. For Southern Sweden, Eckersten [6] has also found earlier harvest dates for winter wheat along with rising temperatures, while the yields stayed the same or decreased.

### Growing period and rainfall during growing period

While comparing the growing period of winter wheat (*V*_per_) in SCEN (1981–2010) with the *V*_per_ in *T*_max_ (2041–2070), there is a reduction of 45 (14 %) days in both regions. These findings correspond with Brown and Rosenberg [3] who calculated the length of the growing season of winter wheat in North America with different GCM. They pointed out that with increasing temperature the potential of water stress may arise. Reciprocal to the growing days we calculated the so-called cold days, with less than 2.5 °C, during the growing period. The amount of cold days decreased by >60 % to 19 days in the *T*_max_ scenario. Walther et al. [19] discovered a comparable trend for frost days when analysing recent data of southern Switzerland. This could be relevant for vernalisation. Porter and Gawith [15] reported the optimal temperature for vernalisation process of winter wheat is between 3.8 and 6.0 °C, while in this study 2.5 °C [18] was taken to define cold days. Further regional adopted climate evaluations have to take care of optimal parameters. Under current conditions, 32 % (DH) to 36 % (OS) of the precipitation within the growing period comes during the main growing period from beginning of hydrological summer to harvest date. We observed a distinct shift of the precipitation towards the period in which the wheat plant does not require a lot of water (sowing until 1 March).

## Conclusion and outlook

It became clear that there is a relevant difference comparing the status quo with current climate projections for the NPG. We found clear indications that the available precipitation during main growing period of winter wheat will decrease. Effects on yield have to be investigated using an appropriated plant soil model. While total annual rainfall does not change significantly a strong shift towards winter precipitation becomes evident. Possible consequences (e.g. nutrient leaching, erosion, need of introduction of catch crops) have to be evaluated in further studies.
